# A novel human 3D lung microtissue model for nanoparticle-induced cell-matrix alterations

**DOI:** 10.1186/s12989-019-0298-0

**Published:** 2019-04-03

**Authors:** Pranita K. Kabadi, April L. Rodd, Alysha E. Simmons, Norma J. Messier, Robert H. Hurt, Agnes B. Kane

**Affiliations:** 1 Department of Pathology and Laboratory Medicine, Brown University, Providence, Rhode Island 02912 USA; 2 AstraZeneca, Gaithersburg, MD 20878 USA; 3 School of Engineering, Brown University, Providence, Rhode Island 02912 USA

**Keywords:** Three-dimensional spheroids, Human lung microtissues, Nanoparticles, Cell-matrix interactions, In vitro testing methods, Multi-walled carbon nanotubes

## Abstract

**Background:**

Multi-walled carbon nanotubes (MWCNT) have been shown to elicit the release of inflammatory and pro-fibrotic mediators, as well as histopathological changes in lungs of exposed animals. Current standards for testing MWCNTs and other nanoparticles (NPs) rely on low-throughput in vivo studies to assess acute and chronic toxicity and potential hazard to humans. Several alternative testing approaches utilizing two-dimensional (2D) in vitro assays to screen engineered NPs have reported conflicting results between in vitro and in vivo assays. Compared to conventional 2D in vitro or in vivo animal model systems, three-dimensional (3D) in vitro platforms have been shown to more closely recapitulate human physiology, providing a relevant, more efficient strategy for evaluating acute toxicity and chronic outcomes in a tiered nanomaterial toxicity testing paradigm.

**Results:**

As inhalation is an important route of nanomaterial exposure, human lung fibroblasts and epithelial cells were co-cultured with macrophages to form scaffold-free 3D lung microtissues. Microtissues were exposed to multi-walled carbon nanotubes, M120 carbon black nanoparticles or crocidolite asbestos fibers for 4 or 7 days, then collected for characterization of microtissue viability, tissue morphology, and expression of genes and selected proteins associated with inflammation and extracellular matrix remodeling. Our data demonstrate the utility of 3D microtissues in predicting chronic pulmonary endpoints following exposure to MWCNTs or asbestos fibers. These test nanomaterials were incorporated into 3D human lung microtissues as visualized using light microscopy. Differential expression of genes involved in acute inflammation and extracellular matrix remodeling was detected using PCR arrays and confirmed using qRT-PCR analysis and Luminex assays of selected genes and proteins.

**Conclusion:**

3D lung microtissues provide an alternative testing platform for assessing nanomaterial-induced cell-matrix alterations and delineation of toxicity pathways, moving towards a more predictive and physiologically relevant approach for in vitro NP toxicity testing.

**Electronic supplementary material:**

The online version of this article (10.1186/s12989-019-0298-0) contains supplementary material, which is available to authorized users.

## Background

The potential of engineered nanomaterials (NM) to enhance performance in a variety of technology sectors has been a major driver for industrial growth and innovation over the last decade. Carbon-based NMs, such as carbon nanotubes (CNTs) and graphene, are particularly attractive for some industrial uses due to their stability and unique geometries and physicochemical properties [1]. Multi-walled carbon nanotubes (MWCNTs) are among the most abundantly produced carbon NMs [2]. While MWCNTs can offer real performance advantages in large-scale applications such as electronic devices and conductive composites, the associated occupational, consumer, and environmental exposures have raised concern regarding potential health risks [1, 3]. The high aspect ratio and fibrous characteristics of MWCNTs, similar to that of asbestos fibers, have been implicated in lung toxicity elicited following exposure in rodent models [4–7]. Several studies have previously documented MWCNT-induced pulmonary toxicity in vivo and in vitro, though the direct implications of these adverse effects for human health are currently under investigation.

Carbon nanotubes have been reported to modulate inflammatory responses and to induce oxidative stress, development of granulomas, and pulmonary fibrosis in rodent lungs [8–13]. Animals exposed to MWCNTs by oropharyngeal aspiration, intratracheal instillation, or inhalation demonstrated acute inflammation with pulmonary infiltration of polymorphonuclear (PMN) leukocytes and other white blood cells into alveolar spaces, and elevated levels of pro-inflammatory cytokines such as IL-1β and IL-6 in bronchoalveolar lavage fluid (BALF) [5, 14, 15]. Furthermore, inflammatory responses were often sustained, contributing to chronic inflammation and the development of subsequent lung pathologies [9, 12, 16–18]. Histopathological and biochemical data from previous studies have shown abnormal deposition of collagen, formation of granulomas, and increased expression of pro-fibrotic factors such as TGF-β1, PDGF-A, osteopontin, and collagen in lung tissue from exposed animals [16–19]. Specific pathways including IL-1β mediated inflammasome activation and TGF-β/SMAD signaling have been implicated in the development of pulmonary fibrosis [20–22]. Microarray analysis of mRNA and miRNA expression following exposure to MWCNTs in mice identified several regulatory networks involved in sustaining a progressive fibrotic phenotype following an initial inflammatory response, including pathways for leukocyte migration, extracellular matrix remodeling, chronic inflammation, and other signaling pathways involved in development of fibrosis [23].

Respirable inhaled particles deposit by impaction and interception in the distal airways, especially in the region of the terminal bronchioles and alveolar duct bifurcations [24]. This deposition pattern was initially observed in rodents following inhalation of asbestos fibers [24] and was recently confirmed following inhalation of multi-walled carbon nanotubes in mice [25]. Lung macrophages are attracted to sites of particle deposition following complement activation [26] where they actively phagocytize inhaled particles [27, 28]. Macrophages mediate particle clearance from the terminal regions of the lungs; however, biopersistent high aspect ratio fibrous particulates like asbestos fibers and multi-walled carbon nanotubes trigger release of proinflammatory mediators and cytokines that recruit additional macrophages and inflammatory cells to sites of particle deposition [25, 27, 29]. Biopersistent particles interact with macrophages, lung epithelial cells, and fibroblasts at discrete anatomic sites called the epithelial-mesenchymal trophic unit [30]. Cooperative interactions between macrophages, lung epithelial cells, and fibroblasts at these anatomic locations lead to persistent inflammation, lung injury and repair, and extracellular matrix deposition initially around the terminal bronchioles that extends into the interstitium [25, 29, 30].

Paracrine signaling between lung macrophages, epithelial cells, and fibroblasts is hypothesized to amplify and sustain the inflammatory response to inhaled nanoparticles leading to lung injury and fibrosis [31]. These cell-cell interactions have been modeled in transwell co-cultures of macrophages and epithelial cells [32] or by transfer of conditioned medium from macrophages or epithelial cells to fibroblasts in vitro [20, 33, 34]. Comparative transcriptomic analyses have detected different gene expression patterns between cells in monolayer cultures compared to mouse lungs following instillation of multi-walled carbon nanotubes [35]. Snyder-Talkington et al. [36] noted greater concordant expression of genes related to inflammation and fibrosis in co-cultures than in monocultures when comparing transcriptomic profiles of mouse lungs exposed to the same sample of carbon nanotubes.

The potential adverse human health impacts of nanomaterials emphasize the need for improved and more efficient nanotoxicology testing platforms relevant to human physiology to enable design and selection of safer nanomaterials in the future [37]. The current standard for nanotoxicology testing utilizes time-intensive in vivo models, severely limiting the number of nanomaterials that can be evaluated for safety, and resulting in a growing catalog of untested nanoparticles [38]. Although two-dimensional (2D) monolayer in vitro models are time and cost efficient, they lack the complexity of intact physiological systems and may not provide results that can be correlated with chronic in vivo responses [38–40]. Existing in vitro lung models have been focused on the use of synthetic scaffold-based systems or transwell models designed to mimic the pulmonary air-liquid-interface (ALI) [40]. These conventional models do not fully recapitulate the complexity of tissue morphology and organization, cell-cell contacts, or chronic responses that require longevity and functionality beyond an acute time frame [41]. Three-dimensional (3D) in vitro platforms provide a unique alternative to bridge the gap between traditional 2D in vitro and in vivo models, allowing for better replication of in vivo tissue function through 3D cell-cell and cell-matrix interactions and prolonged viability [40, 42].

In the current study, a novel 3D lung microtissue platform consisting of human lung epithelial cells, fibroblasts, and macrophages was developed and characterized. Microtissues were exposed to MWCNTs and asbestos fibers known to induce adverse pulmonary responses in vivo. The obtained data demonstrate the potential of 3D human lung microtissues as an alternative model for the assessment of nanomaterial-induced pulmonary toxicity and the subsequent histopathological and molecular changes associated with persistent inflammation and altered cell-matrix interactions.

## Methods

### Materials

Isometric elemental carbon nanoparticles, carbon black M120, were purchased from Cabot Corp. (Boston, MA) and as previously characterized, have a primary diameter of 75 nm and a total surface area of 35 m^2^/g. Multi-walled carbon nanotubes (MWCNT-7) from Mitsui & Co. Ltd. (Tsukuba Ibaraki, Japan) generously provided by Dr. Gunter Oberdörster (University of Rochester, Rochester, NY), and crocidolite asbestos fibers, purchased from Duke Scientific (Palo Alto, CA), were also previously characterized for size distribution and surface area [43]. The physiochemical properties of these materials are summarized in Table 1.

**Table 1:** Physicochemical characterization of nanomaterials

Sample and Origin	Length (μm)	Diameter (nm)	Fe content (wt- %)	Surface area (m^2^/g)
Carbon Black M120 Cabot Corp.	N/A	75	ND	35
MWCNT-7^a^ Mitsui & Co.	11.7 ± 3.6	80 ± 18	< 0.2	20
Crocidolite asbestos^a^ UICC	2.8 ± 2.6	116 ± 112	22.2	9

*ND* Not detectable, *N/A* Not applicable

^a^As reported in Sanchez et al., 2011.

To ensure sterility and maintenance of an endotoxin-free environment, stock suspensions of materials were conducted using sterile methods under a Class IIB biological safety hood with external exhaust. Stocks for M120 carbon black and MWCNT-7 were suspended in sterile, endotoxin-free PBS containing 10% dipalmitoylphosphatidylcholine (DPPC) and 3% bovine serum albumin (BSA) (Sigma-Aldrich, St. Louis, MO) and sonicated for 45 min in an ultrasonic bath sonicator (Branson Ultrasonic Corporation, Danbury, CT) to ensure nanomaterial dispersion before dilutions to 1000 μg/mL in RPMI medium (Life Technologies, Grand Island, NY) containing 1% BSA (Sigma-Aldrich, St. Louis, MO) and the production of subsequent working stocks in complete cell culture media. Working stocks of both nanomaterials were sonicated again prior to exposure of cell cultures. This dispersion technique produced uniform suspensions of individual particles or small agglomerates of carbon nanotubes as described previously [43].

Crocidolite asbestos fibers were baked at 250 °C as previously described and re-suspended in sterile, endotoxin free PBS to generate a stock solution of 1000 μg/mL [43]. Asbestos stocks were diluted to produce working stocks in complete cell culture media for exposures. Endotoxin levels in the stock particle suspensions were determined using the gel-clot assay and were less than 0.31 EU/mL (Associates of Cape Cod, Inc.).

### Lung cell monolayer cultures

Target cell lines were selected to model the anatomic site of initial particle-cell interactions following deposition in the terminal bronchioles [24]; BEAS-2B lung bronchial epithelial cells and THP-1 human monocytic cells were selected because they have been widely used for nanotoxicology assays using harmonized exposure protocols [44]. BEAS-2B immortalized human lung bronchial epithelial cells (ATCC, Manassas, VA) were cultured in BEGM media with SingleQuots supplement kit (Lonza, Basel, Switzerland) at 37 °C in 5% CO_2_. THP-1 human monocytic cells (ATCC, Manassas, VA) were cultured using RPMI-1640 (Gibco) with 10% heat-inactivated fetal bovine serum (FBS) (Atlanta Biologicals, Flowery Branch, GA) and 1% penicillin/streptomycin at 37 °C and 5% CO_2_ in 100 mm low-attachment cell cultures dishes. Prior to use, THP-1 cells were differentiated into macrophages with 10 nM phorbol 12-myristate 13-acetate (PMA) (Fisher Scientific, Agawam, MA) for 72 h. IMR-90 human lung fibroblasts, generously provided by Dr. Anatoly Zhitkovich (Brown University, Providence, RI), were maintained using DMEM high glucose (Gibco) with 10% FBS and 1% penicillin/streptomycin at 37 °C in 5% low O_2_ conditions and 5% CO_2_. Cells were collected and washed in fresh medium prior to nanomaterial exposure. All cell lines were used before passage 20.

### 3D cell culture and microtissue exposure

To culture optimized 3D lung microtissues, 2% agarose hydrogels [45] created from small spheroid micromolds (Microtissues, Inc., Providence, RI, No. 24–96 or 12–256) were first equilibrated for 30 min in DMEM high glucose supplemented with 10% FBS and selected Single Quot supplements (the Lonza BEGM SinqleQuots™ supplement kit with bovine pituitary extract (BPE) and human epidermal growth factor (hEGF) excluded). Prior to seeding the hydrogels, THP-1 macrophages were primed with 100 ng/mL lipopolysaccharide (LPS) (Sigma-Aldrich, St. Louis, MO) for 4 h. Primed THP-1 cells were pre-exposed to particles for 3 h, then combined with IMR-90 fibroblasts and BEAS-2B epithelial cells to form a single cell suspension. A total of 81,000 cells were suspended in DMEM high glucose medium (Gibco) with 10% FBS and seeded per 256-spheroid hydrogel in triculture medium (yielding 316 cells per microtissue). After 24 h, medium was replaced with DMEM high glucose medium (Gibco) plus 0.5% FBS with SingleQuots™ (BPE and hEGF excluded) and maintained in this triculture medium until collection [39]. During the optimization process, these microtissue culture conditions were modified to determine the effects of different media conditions, seeding density, and cell ratios as described in Additional file 1: Figure S1. A 4:1:1 ratio of primed THP-1 macrophages to epithelial cells and fibroblasts was selected to mimic initial recruitment of circulating leukocytes from the blood into the lungs following inhalation of particles or fibers [27]. Quantitative morphometric analyses in rodents following inhalation of asbestos fibers revealed a 3 to 10-fold increase in lung macrophages two days after exposure [29].

### THP-1 differentiation, priming, and characterization

To evaluate macrophage phagocytosis, 24-well glass bottom plates (Eppendorf 0030741021) were coated with poly-L-lysine (Sigma-Aldrich, St. Louis, MO) according to manufacturer’s protocol. Two hundred thousand macrophages were plated in the poly-L-lysine-coated wells in media containing LPS and nanomaterials in 200 μL per well. Cells were incubated at 37 °C and 5% CO_2_ for 4 h to complete priming. After priming, medium was removed and replaced with DMEM high glucose medium (Gibco) plus 10% FBS with SingleQuots™ (BPE and hEGF excluded) containing Hoechst 33342 (Thermo Fisher Scientific). To visualize release of cathepsin B from lysosomes, cells were incubated with Bio-Rad Magic Red™ Cathepsin B substrate followed by Hoechst 33342 (Thermo Fisher Scientific) for 10 min before imaging using an Olympus confocal microscope (Additional file 1: Figure S1).

### Confocal fluorescence imaging of 3D microtissue assembly

To image 3D microtissue assembly and quantitate volumetrics, cells were stained with either CellTracker Deep Red, CellTracker Green CMFDA, or CellTracker Orange CMRA (Molecular Probes/Thermo Fisher Scientific) using the manufacturer’s protocol prior to assembly into microtissues. The labeled cells were imaged in live microtissues using confocal fluorescence microscopy. For volumetric analysis, live microtissues were imaged using quantitative high content imaging (Opera Phenix, Perkin Elmer) and Harmony analysis software was used to identify each fluorescently-labeled cell population. Z-stacks of intact microtissues were obtained using confocal laser scanning fluorescence microscopy throughout the depth of the microtissue. Microtissue volume was calculated based on analysis of 142, 177, and 96 microtissues on days 2, 4, 7, respectively.

### Microtissue histology

For histological evaluation, 3D lung microtissues were fixed in 10% neutral buffered formalin (Fisher Scientific) in agarose hydrogels for a minimum of 24 h prior to further processing. Microtissues were then processed for embedding in Technovit 7100 glycol methacrylate (Electron Microscopy Sciences, Hatfield, PA, Heraeus Kulzer GmBH) as described previously [39]. Following embedding in glycol methacrylate, samples were cut in 3 μm sections, mounted on slides, and stained with hematoxylin and eosin (H&E) for histological examination using bright field light microscopy.

### Microtissue viability

Microtissue viability was assessed using the CCK-8/WST-8 cell viability assay (Dojindo Molecular Technologies, Inc., Rockville, MD) following exposure for 4 and 7 days. The manufacturer’s protocol was slightly modified to enable assays using 3D microtissues. Media surrounding the hydrogels was carefully aspirated without disturbing the microtissues within. The remaining media in the top seeding chamber of the hydrogels was removed gently by pipetting, after which 150 μL of fresh media containing CCK-8 solution was added to the top seeding chamber and incubated for 3 h at 37 °C in 5% CO_2_. After 3 h, 100 μL of solution was removed from each hydrogel and transferred to a 96-well plate and read for absorbance as per manufacturer’s recommendations. Statistical analyses were based on mean values obtained in three independent experiments. Significance was calculated by comparing between viability at each dose in comparison to untreated microtissues.

### RNA isolation, PCR arrays, and qRT-PCR assays

To collect RNA from microtissues, 3D lung microtissues were centrifuged out of the agarose mold, at 1000 rpm for 5 min at 4 °C, collected into conical tubes, and rinsed in PBS twice. Samples were lysed with TRI Reagent (MRC, Cincinnati, OH) and stored at − 80 °C. Total RNA was then isolated as described previously [46]. For analysis of gene expression using Human Fibrosis RT^2^ Profiler PCR Arrays (PAHS-120Z), DNase treated RNAs isolated from 3D microtissues were cleaned through RNeasy® MinElute™ columns, then converted to cDNAs and used for analysis according to vender specifications (Qiagen, Germantown, MD). Resulting data were uploaded to the Qiagen web portal (www.qiagen.com), normalized to averaged reference transcripts with differences in C_T_ values < 1 across all sample groups, and evaluated using ΔΔC_T_ analysis (array data for 4 day exposure in Additional file 2, and 7 day exposure in Additional file 3). Genes with statistically significant gene expression changes and/or changes greater then 2-fold when compared to untreated microtissues were displayed in heat map format and a subset of the significant changes (statistically significant and greater than 2-fold change) are shown in Venn diagrams (selected array data listed in Additional file 4).

### Protein isolation and Luminex bead assays

3D microtissue samples were collected by centrifugation at 1000 rpm for 5 min at 4 °C and rinsed twice with PBS as described above for RNA isolation. Pelleted samples were lysed on ice in PBS containing 0.1% Triton X-100, 1 mM EDTA, and cOmplete™ Protease Inhibitor Cocktail (Millipore Sigma, USA) followed by centrifugation to remove cell debris. The total protein concentration of each sample (mg/mL) was determined using a Bio-Rad DC Protein Assay kit (Bio-Rad, USA). For Luminex (USA) bead assays, 3-plex (MMP1, MMP2, MMP3) and 4-plex (IL-1β, IL-6, HGF, TNF-α) magnetic bead kits, including reagents, detection antibodies, and standards, were assembled by mixing pre-coupled magnetic beads selected from Bio-Rad’s Pro Human Inflammation (Cat#171-AL001M) and Human Cytokine Screening (Cat# 12007283) multiplex panels. All protein samples were assayed in duplicate using kit protocols supplied by the manufacturer. Data collection was performed on a Bio-Rad BioPlex 200 plate reader employing BioPlex Manager software. Final analyte levels (pg/mL) were corrected for total protein in each sample and reported as picogram of analyte per milligram of total protein.

### Statistical analyses

All data are reported as mean ± standard deviation (SD) unless otherwise stated. Volumetric, viability, and qRT-PCR data were analyzed using one-way ANOVA with Dunnett’s multiple comparisons test using GraphPad Prism 6 software. Each exposure was compared to untreated microtissues to yield multiplicity adjusted *p*-values, which are reported in the figures. Statistical analysis of viability data was performed across doses compared to untreated microtissues for each nanomaterial separately. For MMP qRT-PCR assays, changes in gene expression following exposure to each of the test nanomaterials were compared to untreated microtissues (*) or compared to the same dose across all nanomaterials (#) using one-way ANOVA with Dunnett’s multiple comparisons test. Using R statistical software, raw data (Ct) values for the PCR lung fibrosis array were imported, normalized to obtain ΔCt values, normalized to the reference gene (HPRT1), and analyzed for statistically significant differences between groups using adjusted significance *p*-values for multiple experiments (q-values). The ΔCt values were also used to calculate p-values using Student’s t test. All statistically significant results were identified by *p* <  0.05 or *q* <  0.05.

## Results

### Formation of 3D lung microtissues

**Fig. 1:**
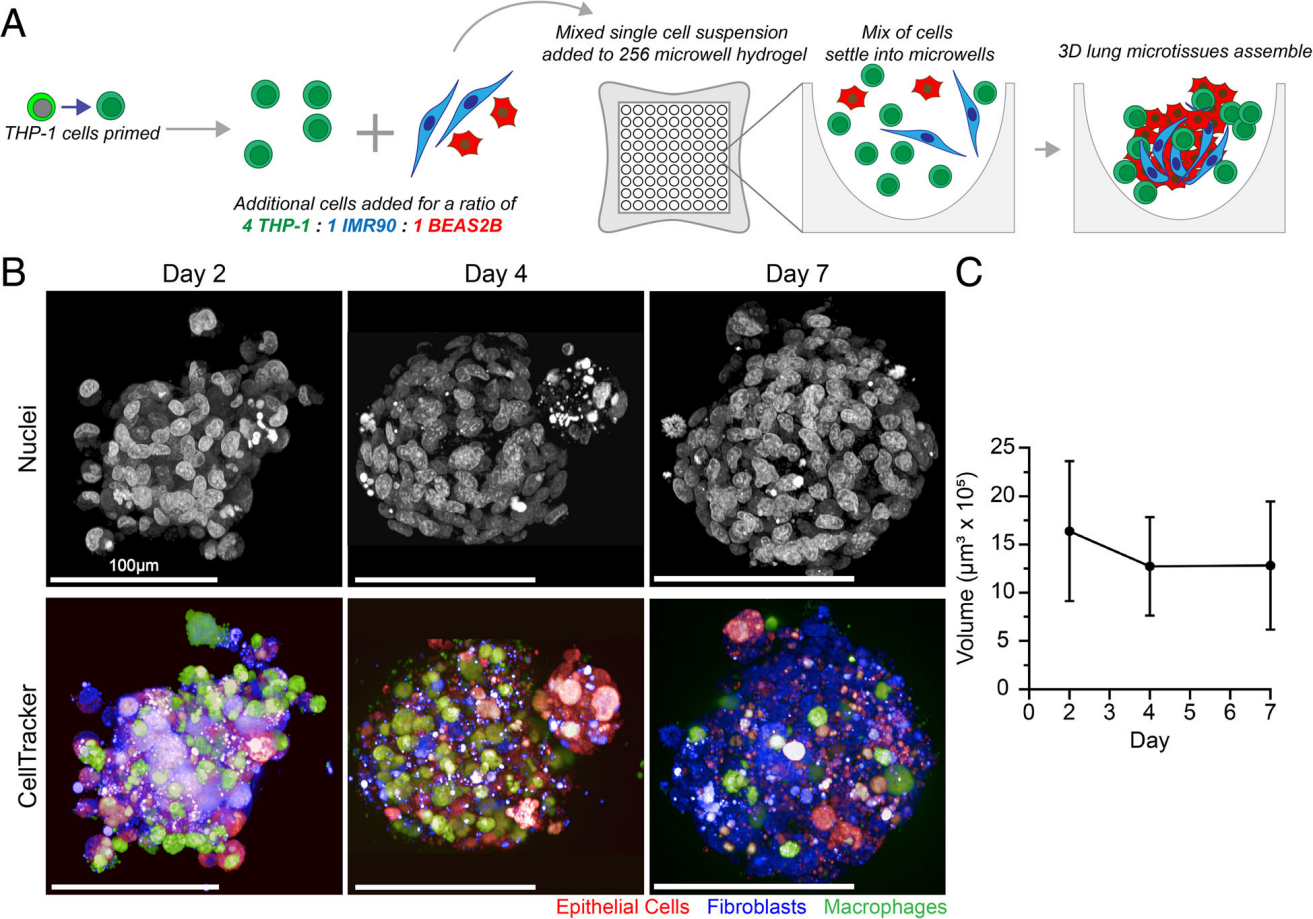
Assembly of 3D human lung microtissues. **a** 3D lung microtissues are plated from a single-cell suspension of three cell lines (BEAS-2B, IMR-90, and THP-1) into a 256-microwell hydrogel. The single cell suspension is seeded into each hydrogel, settles by gravity into the microwells, and cells self-assemble to form microtissues. **b** Individual cells labeled with fluorescent CellTracker dyes were imaged using confocal microscopy (shown as maximum intensity projections). Scale bar = 100 μm. **c** Three-dimensional image analysis was used to quantify microtissue volume after 2, 4, and 7 days. Microtissue volume was calculated by averaging the volumes of 142, 177, and 96 microtissues on days 2, 4, 7, respectively. Changes in microtissue volume over time were not statistically significant

Inhaled particles and fibers are engulfed by macrophages and trigger an acute inflammatory response leading to recruitment of additional inflammatory cells, sustained inflammation and injury, and chronic pulmonary fibrosis [27, 31, 47, 48]. The initial step that triggers this cascade of pathological reactions is hypothesized to be lysosomal membrane permeabilization following phagocytosis resulting in intracellular release of cathepsin B and secretion of mature IL-1ß, a potent proinflammatory cytokine [49–51]. This initial triggering step leading to NLRP3 inflammasome activation [52] has been widely assessed using in vitro assays based on the human monomyelocytic leukemia cell line, THP-1 [44, 49, 53]. The THP-1 cell line is representative of monocytes that grow in suspension; in response to PMA exposure, these cells differentiate into macrophage-like cells capable of phagocytosis; a second priming step using LPS is used to initiate transcription of pro-IL-1ß based on harmonized protocols established by the NIEHS Nano GO Consortium [44]. Prior to assembly of this human 3D lung microtissue model, we optimized the dose and exposure time for PMA to obtain stable differentiation, particle phagocytosis, and viability of THP-1 cells (Additional file 1: Figure S1). As recommended by Park et al. [54], we used a lower dose of PMA, 10 nM for 72 h prior to LPS priming and particle exposure (Fig. 1a). Using this optimized protocol, we confirmed that exposure to high aspect ratio fibrous particles, crocidolite asbestos fibers and carbon nanotubes, but not spherical carbon black particles, caused lysosomal membrane permeabilization and intracellular release of cathepsin B using differentiated THP-1 cells as reported previously [49, 55].

3D lung microtissue formation and culture conditions were optimized to sustain balanced growth of the different cell types and maintain viability over extended culture times (Additional file 1: Figure S2). Different ratios of the three cell lines were tested ranging from 1:1:1 to 4:1:1 (THP-1:BEAS-2B:IMR-90) to demonstrate that high numbers of THP-1 macrophages can be incorporated into the structure without preventing microtissue formation (Additional file 1: Figure S2). A ratio of 4:1:1 was chosen to reflect the relatively high proportion of macrophages present in damaged profibrotic and fibrotic rodent lungs following instillation of carbon nanotubes [14]. Both the size and organization of the microtissue were dependent on the initial seeding density as well as media composition (Additional file 1: Table S2). When plated at fewer total cells per spheroid, relative proliferation of the three cell lines enabled one cell type to dominate the microtissue and create a simpler spheroidal microtissue structure (Additional file 1: Figure S2B). Plating at 316 cells per microtissue created a viable spheroid that maintained a complex microtissue structure up to 7 days. Additionally, when cultured in unmodified BEAS-2B culture media, cells continued to proliferate and the microtissues developed a necrotic center by day 7 (Additional file 1: Figure S2C). Exclusion of both bovine pituitary extract (BPE) and human epidermal growth factor (hEGF) supplements was identified to better control microtissue growth and prevent the formation of necrotic centers while maintaining overall organization of the microtissue.

**Fig. 2:**
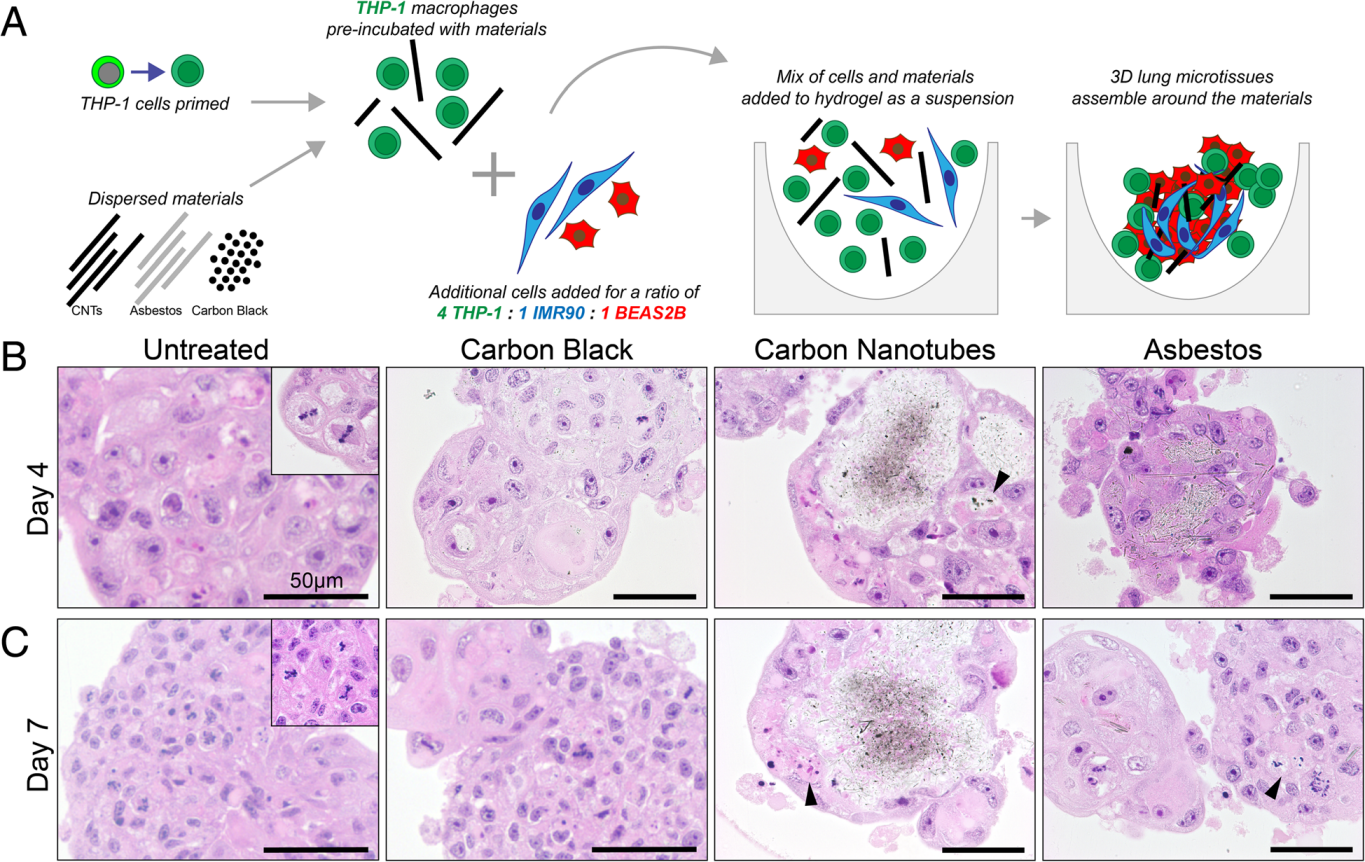
Morphological changes induced by exposure to nanomaterials in 3D human lung microtissues. **a** Primed THP-1 cells are exposed to nanomaterials prior to mixing with fibroblasts and epithelial cells as described in the Materials and Methods. Histopathology of 3D lung microtissues sections following exposure to nanomaterials for 4 days (**b**) or 7 days (**c**). Mitotic figures can be seen in untreated microtissues after 4 and 7 days, as illustrated in image insets. Microtissues exposed to carbon nanotubes have altered microtissue morphology, with large nanoparticle aggregates. Focal cell death (black arrowheads) was observed following exposure to carbon nanotubes and asbestos fibers. Sections were stained with hematoxylin and eosin. Scale bar = 50 μm

Human 3D lung microtissues formed using the optimized culture method developed a variable but ordered structure over time. All three cell types self-assembled into a microtissue (Fig. 1), forming from an initial clustering of fibroblasts and epithelial cells into a spheroid after 2 days (Fig. 1b). After 2 days, microtissues showed BEAS2B epithelial cells intercalating into IMR-90 fibroblast cells, with THP-1 macrophages at the periphery (Fig. 1b). Histological sections of the microtissues revealed assembly of these three cell types into a stable 3D structure. By day 4 each microtissue typically consisted of a single round or multi-lobed spheroid (Fig. 2b). This morphological structure was maintained for at least 7 days (Fig. 2c). Using fluorescence imaging, the volume of microtissues was calculated using 3D image rendering and spheroid surface identification using Harmony software. The average volume of 3D lung microtissues did not statistically change from day 2 to day 7 (Fig. 1c). However, signs of proliferation including mitotic figures were observed in untreated microtissues, indicating that growth was sustained for at least 7 days (Fig. 2). These proliferating cells were identified as epithelial cells using the CellTracker assay (data not shown).

### Nanomaterial exposure and lung microtissue pathology

**Fig. 3:**
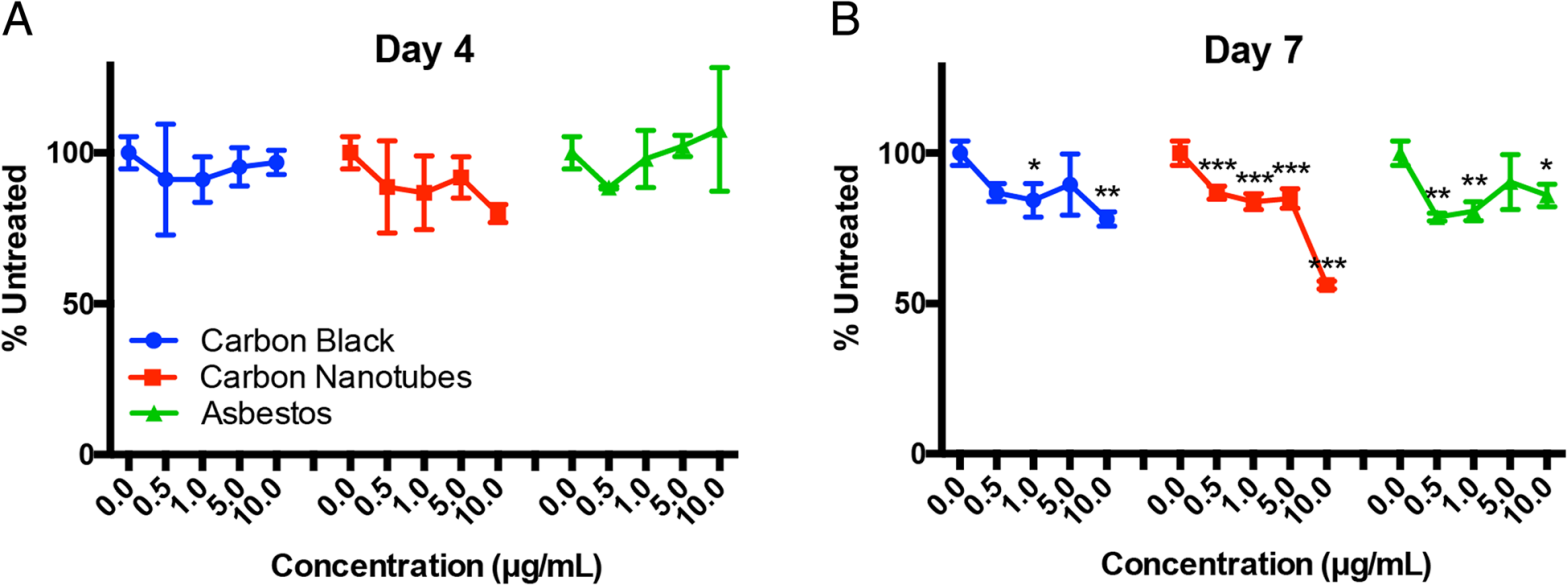
Microtissue viability following exposure to nanomaterials. Viability was assessed using the WST-8 assay after 4 days (**a**) or 7 days of exposure (**b**) to 0.5–10 μg/mL of the test nanomaterials. * *p* < 0.05, ** *p* < 0.01, ***p < 0.001 relative to untreated microtissues. (*N* = 3)

Nanomaterial doses of 0.5–10 μg/mL were selected based on previously published experiments investigating toxicity of inhaled carbon nanotubes (Table 2) [25]. While there was no significant decrease in viability on day 4, microtissues exhibited dose-dependent toxicity by day 7 (Fig. 3). MWCNTs caused the greatest toxicity, with a more than 40% decrease in viability at the highest dose on day 7.

**Table 2:** Nanomaterial dosimetry

3D Lung Microtissue Dose (μg/mL)	Dose per hydrogel (μg)	Dose per Microtissue (ng)
10.0	1.80	7.03
5.0	0.90	3.52
1.0	0.18	0.70
0.5	0.09	0.35

Each hydrogel chamber forms 256 microtissues, and the dose in μg was delivered in a 190 μL volume. The dose for each microtissue is expressed as ng delivered to a total of 316 cells in each microtissue.

Histological assessment of 3D lung microtissues after 4 and 7 days of nanomaterial exposure showed specific morphological changes induced by this panel of nanoparticles. While the well-dispersed carbon black nanoparticles appear internalized by cells and evenly distributed throughout the lung microtissues, most of the MWCNTs were bundled into large regions of agglomerated material (Fig. 2). Smaller MWCNT agglomerates or individual tubes were also scattered throughout the spheroid and some appeared to be internalized within individual cells. While asbestos fibers did agglomerate after exposure at higher doses, shorter fibers were internalized by individual cells resulting in a different morphological distribution than seen following exposure to MWCNTs. Exposure to MWCNTs also induced cell death in cells surrounding the MWCNT agglomerates (Fig. 2). Cell death was observed on day 4 and 7 and was primarily located adjacent to the MWCNTs themselves. Focal cell death was observed in cells in direct contact with asbestos fibers, but overall toxicity is lower than in MWCNT-exposed microtissues. The initial suspension of test particles was well-dispersed and secondary agglomeration of carbon nanotubes in 3D granulomas was reported previously [43]. Untreated lung microtissues did not show cell death at either time point (Fig. 2).

### Induction of inflammatory cytokines and extracellular matrix biomarkers in 3D lung microtissues

**Fig. 4:**
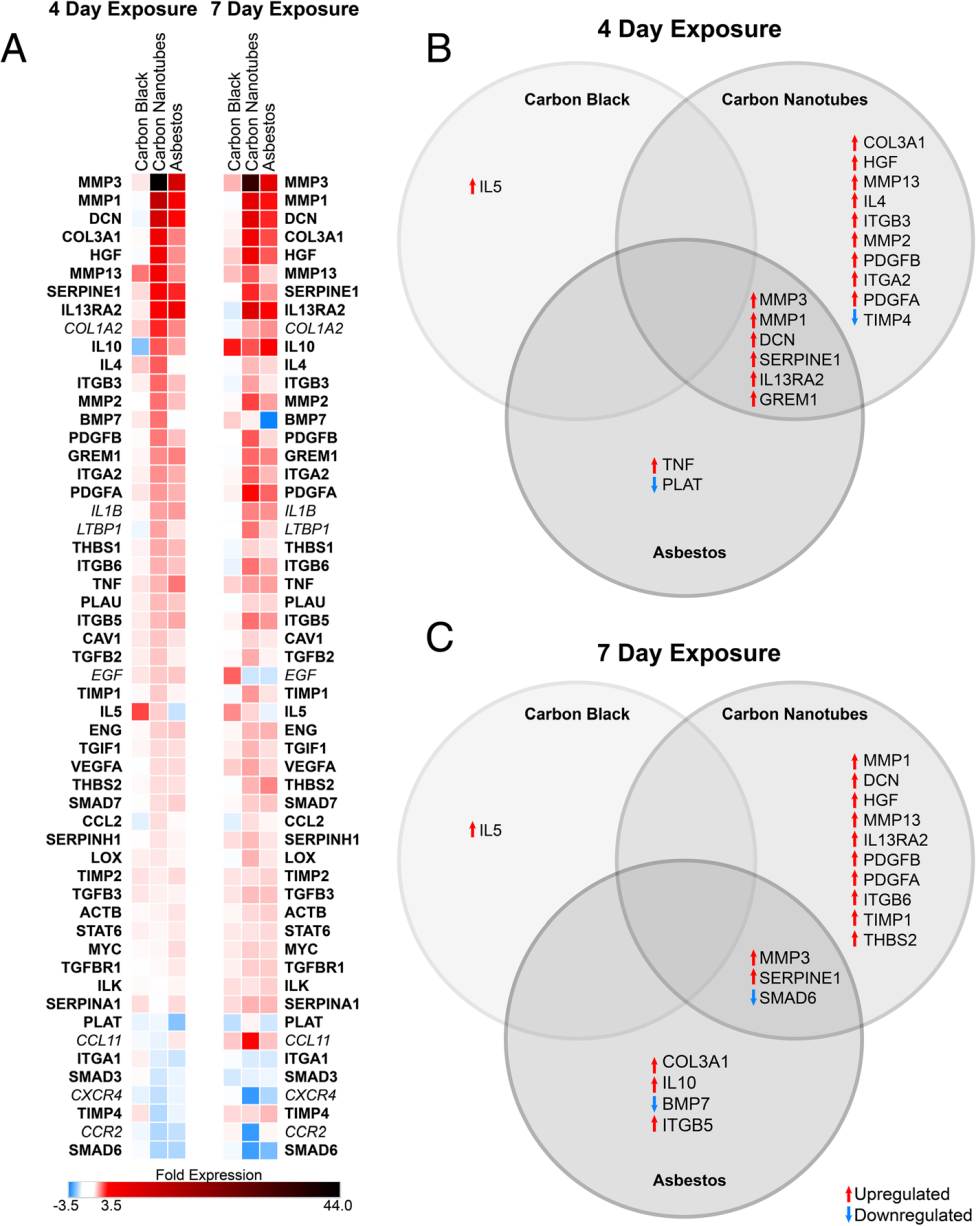
Gene expression profiles in 3D lung microtissues after exposure to 10 μg/mL of test nanomaterials. **a** Gene expression in 3D lung microtissues exposed to 10 μg/mL carbon black, carbon nanotubes, or asbestos fibers for 4 or 7 days was quantified using PCR arrays. Both significant (*p* or q < 0.05, bold) and highly changed non-significant (italics) genes are included in the heat map. Significantly altered gene expression with a more than 2-fold change is shown as Venn diagrams organized by nanomaterial exposure after 4 days (**b**) and 7 days (**c**)

Human 3D lung microtissues were evaluated using a PCR array containing key genes and pathways associated with inflammation and cell-matrix interactions (Fig. 4, Additional file 1: Figure S3). RNA was isolated from microtissues after 4 and 7 days of exposure to 10 μg/mL carbon black, MWCNTs, or asbestos fibers, then used for quantitative analysis of gene expression. Genes significantly changed (*p* or q < 0.05, or *p* > 0.05 with higher than a 2-fold change in expression) in at least one treatment group at either time point are highlighted using a heat map to show changes relative to untreated 3D lung microtissues (Fig. 4a). These statistically significant alterations in gene expression include inflammatory cytokines, fibrotic mediators, extracellular matrix proteins, matrix metalloproteases (MMPs), and TGF-β signaling previously associated with MWCNT-induced lung pathologies [30, 31, 56]. Upregulated genes included extracellular matrix components (collagens and decorin), cytokines, growth factors, and MMPs and their inhibitors. The most significantly altered genes (*p* or q < 0.05, with greater than or equal to 2-fold change in expression) showed that differential expression of these genes is exposure specific (Fig. 4b and c). After 4 days of exposure, 3D lung microtissues exposed to MWCNTs showed induction of expression of several genes that were subsequently elevated after 7 days following asbestos exposure. Several highly upregulated genes, including *MMP1*, *MMP3*, and decorin (*DCN*), were induced by both asbestos and MWCNT exposure. This pattern continues at day 7, with MWCNT exposure resulting in the most significant changes in gene expression (Fig. 4b and c). While MWCNTs and asbestos induced several of the same genes, induction of these genes was not observed in microtissues exposed to carbon black.

**Fig. 5:**
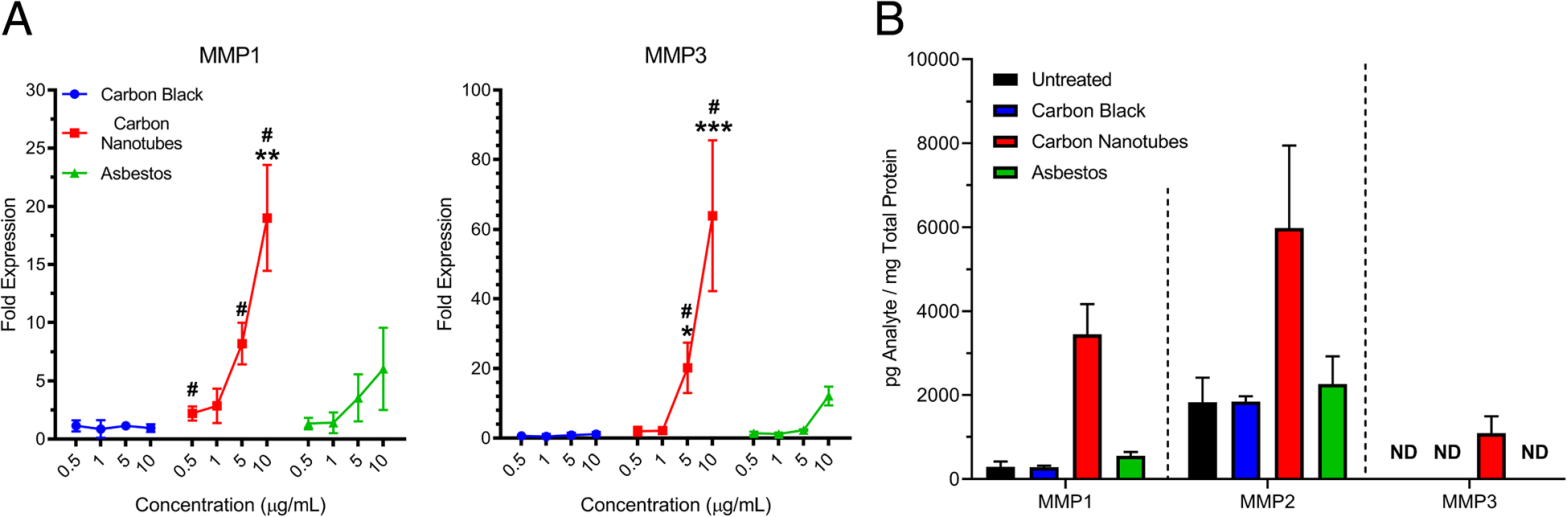
MMP gene and protein expression induced by exposure of 3D lung microtissues to test nanomaterials for 4 days. **a** Dose response of MMP1 and MMP3 gene expression analyzed using qRT-PCR. Statistical significance is indicated as *p < 0.05, **p < 0.01, and ***p < 0.001 for comparisons between each dose relative to untreated microtissues. Statistical significance is indicated by ^#^*p* < 0.05 for comparisons between the same dose of each of the three nanomaterials relative to untreated microtissues. **b** Expression of MMP proteins in microtissue protein lysates collected after exposure to test nanomaterials for 4 days. These experiments were repeated twice. *ND* = not detectable

Dose-dependent expression of several key genes was confirmed using qRT-PCR (Fig. 5). Expression of collagen type I (*COL1A1*), which was not included on the fibrosis PCR array, collagen type 3 (*COL3A1*), and decorin, a proteoglycan in extracellular matrix, was increased by exposure to carbon nanotubes for 4 days (Table 3). *MMP1* and *MMP3* were the most highly upregulated genes in MWCNT-exposed microtissues on days 4 and 7. qRT-PCR analysis exhibited a dose-dependent increased expression of *MMP1* and *MMP3* in asbestos-exposed microtissues, but statistically significant increases were only observed after exposure to carbon nanotubes. These results were confirmed by increased protein expression of MMP1, MMP2, and MMP3 in lysates of 3D microtissues following exposure to 10 μg/mL carbon nanotubes for 4 days (Fig. 5b).

**Table 3:** Gene expression of selected extracellular matrix components in 3D lung microtissues using qRT-PCR

ECM Protein Mean Fold Change ± SD	Carbon Black	Carbon Nanotubes	Asbestos
COL1A1	1.12 ± 0.23	3.64 ± 0.82	2.12 ± 0.51
	*p* = 0.981	*p* = 0.005	*p* = 0.062
COL3A1	1.33 ± 0.15	6.33 ± 0.65	2.57 ± 0.25
	*p* = 0.555	*p* < 0.001	*p* = 0.002
Decorin	0.97 ± 0.04	10.97 ± 1.58	3.53 ± 1.69
	*p* = 0.999	*p* < 0.001	*p* = 0.065

Microtissues were exposed to 10 μg/mL of each test nanomaterial for 4 days. qRT-PCR was used to confirm the relative gene expression changes detected in the PCR arrays (Fig. 4). Mean fold changes in gene expression were calculated relative to untreated microtissues based on 3 independent experiments. A *p*-value < 0.05 is considered statistically significant.

**Fig. 6:**
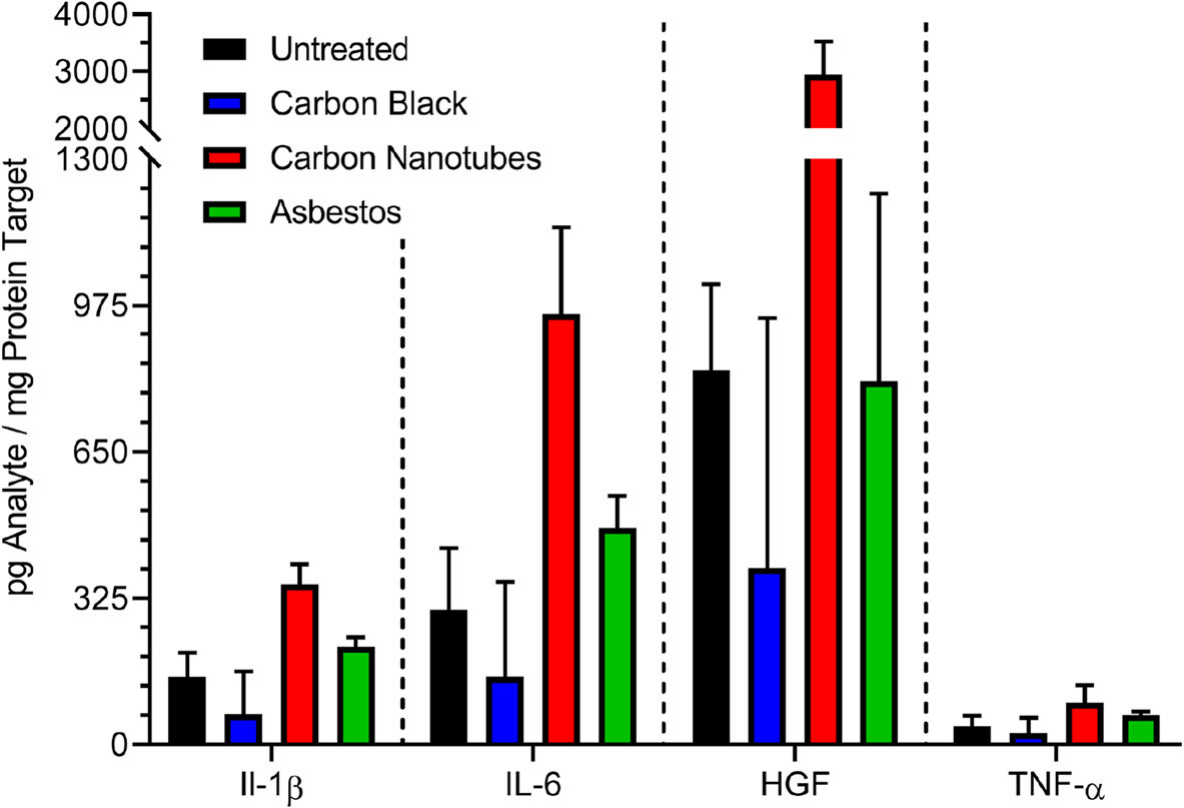
Cytokine protein expression in 3D microtissues exposed to test nanomaterials. Microtissue lysates were analyzed for expression of cytokines (IL-1β, IL-6, TNF-α) or the growth factor, HGF, following exposure to 10 μg/mL of carbon black, carbon nanotubes, or asbestos fibers for 4 days. Mean analyte contents per mg of total protein in the lysate were quantified using Luminex bead assays as described in Materials and Methods. The mean is based on duplicate experiments

Upregulation of selected cytokine genes detected in the PCR arrays (Fig. 4) was also confirmed using protein quantification in microtissue lysates following exposure to 10 μg/mL of test nanomaterials for 4 days (Fig. 6). An increased level of IL-1β was detected in microtissues exposed to carbon nanotubes and asbestos fibers consistent with lysosomal membrane permeabilization and release of cathepsin B (Additional file 1: Figure S1) indicative of inflammasome activation as reported previously in primed THP-1 cells [49, 53]. Increased levels of the proinflammatory cytokines, TNF-α and IL-6, were also detected following exposure to carbon nanotubes or asbestos fibers. Finally, hepatocyte growth factor (HGF) was also detected in 3D microtissues exposed to carbon nanotubes.

## Discussion

The 3D lung microtissues evaluated in the current study are complex, three-dimensional structures that contain the cell types representative of the epithelial-mesenchymal trophic unit involved in persistent inflammation, lung injury, aberrant repair, and fibrosis [30, 31]. This toxicity testing platform is proposed as a bridging technology between in vitro and in vivo toxicity studies with the goal to reduce animal testing to assess potential toxicity of nanomaterials [57]. Other in vitro lung models have been developed, but have not been used to assess persistent inflammation and cell matrix interactions predictive of pulmonary fibrosis [58–62]. These alternative models include scaffold-free 3D cell culture, acellular lung scaffolds [63], air-liquid interface models [58–61], and lungs-on-a-chip [64, 65].

In contrast to these alternative testing platforms, this novel 3D lung microtissue model incorporates three human cell lines representing lung epithelial cells (BEAS-2B), fibroblasts (IMR-90) and differentiated macrophages (primed THP-1 monocytes). Microtissues were assembled at cell ratios designed to mimic the morphological changes previously observed in rodents exposed to fibrogenic materials [29, 66, 67]. By pre-incubating differentiated macrophages with nanomaterials [68], we mimic the initial inflammatory cell influx and activation induced by inhalation of toxic particles [27, 59, 69, 70]. This 3D microtissue model promotes paracrine interactions between macrophages, lung epithelial cells, and fibroblasts leading to persistent inflammation and cell-matrix alterations induced by exposure to high aspect ratio fibrous particles [31]. Most in vitro studies that assess toxicity of carbon nanotubes use single cell lines and cell-specific endpoints, excluding the effects of paracrine cytokine signaling or extracellular matrix proteins on neighboring cell types [43, 56, 71]. Microfluidic lungs-on-a-chip have been used to evaluate pulmonary inflammatory responses for 3–5 weeks while also introducing shear forces and alveolar gas exchange, but they do not include immune cells or fibroblasts [72]. A previous study utilized a similar scaffold-free 3D platform to investigate the ability of high-aspect ratio MWCNTs to induce macrophage epithelioid granulomas after longer exposures using only cultured murine-derived primary bone marrow macrophages [43]. While these models have the ability to recapitulate specific features of nanomaterial toxicity, the lack of multicellular complexity in both the 3D granuloma and lung-on-a-chip models limits their ability to study the pathogenesis of pulmonary fibrosis and other chronic pulmonary diseases [14, 17]. Multicellular paracrine interactions and cell-cell contacts are critical for development of structural alterations that occur in the epithelial-mesenchymal trophic unit following inhalation of fibrous nanomaterials [53, 73]. While this 3D lung microtissue platform captures inflammatory endpoints and extracellular matrix alterations in response to carbon nanomaterials, it also has limitations. Specific cell types, like type I and II alveolar cells and other immune cells, are not included in the model. As with any in vitro model, lung microtissues also lack the cross-talk between other organ systems that contribute to systemic inflammatory responses [74, 75]. Unlike in vivo exposures, the 3D lung microtissue lacks clearance mechanisms which may increase the sensitivity of this model to biopersistent materials.

Morphological and molecular alterations induced by this panel of test particles were assessed after 4 to 7 days of exposure. Upregulated expression of collagen genes 1A1 and 3A1 was detected following exposure to MWCNTs; however, MMP genes and proteins were also upregulated. MMPs are involved in extracellular matrix breakdown and remodeling and liberate growth factors bound to matrix components [76]. At these time points, we were not able to detect mature, cross-linked collagen in 3D microtissues exposed to MWCNTs or asbestos fibers using immunohistochemistry. In skin fibroblast monolayers, mature collagen matrix was only observed 18 days after exposure to ascorbic acid [77]. These 3D microtissues were not supplemented with ascorbic acid that is required for post-translational modifications and cross-linking of types I and III collagen. Decorin, a proteoglycan that binds extracellular collagen as well as TGF-β [78], was also upregulated, especially after exposure to MWCNTs. In addition, upregulated hepatocyte growth factor (HGF) gene and protein expression was detected following exposure to MWCNTs. This growth factor is produced by mesenchymal cells and is increased in several models of lung injury where it promotes survival of lung epithelial cells and decreases myofibroblast accumulation and ECM deposition [79]. Longer exposure times to MWCNTs or asbestos fibers in 3D microtissues supplemented with ascorbic acid may be required to promote differentiation and survival of myofibroblasts leading to collagen accumulation [31].

While the complexity of the 3D lung microtissue model presents a challenge to increasing throughput, it is a necessity for quantification of complex endpoints predictive of chronic adverse outcomes like fibrosis. To calibrate the microtissue response, we exposed 3D human lung microtissues to crocidolite asbestos fibers known to induce lung fibrosis after chronic exposure in rodents as well as in human epidemiological studies [67]. Low surface area carbon black nanoparticles were used as a reference sample that has been shown to induce minimal toxicity in rodent inhalation studies and closely matches the surface area of the MWCNTs used in this study [4, 43]. Microtissues were evaluated using a combination of histopathology and molecular biomarkers to combine a targeted approach with morphological endpoints. After 4 and 7 days, long and rigid asbestos-like MWCNTs induced a distinctive morphological change in the microtissues that was not observed following exposure to non-fibrous carbon nanoparticles used as a negative reference sample. These test materials did not impact the overall assembly kinetics of the microtissues. In contrast to the more readily dispersed crocidolite asbestos, carbon nanotubes undergo secondary agglomeration in the lung microtissues that may be the result of their surface hydrophobicity [80]. This “bundling” response to carbon nanotubes is histologically similar to the formation of granulomas or fibrotic foci in the lung after inhalation exposure [17]. While toxicity is observed in cells in direct contact with the MWCNT bundles, it is unclear if the agglomeration or bundling process impacts the changes induced in the microtissue overall [53].

The canonical TGF-β1 pathway is considered to be a critical determinant of matrix deposition in the airways and lung interstitium leading to progressive pulmonary fibrosis, regardless of the initial insult [18, 30, 81]. Exposure to carbon nanotubes has also been shown to activate additional signaling pathways leading to fibrosis including non-canonical TGF-β1 signaling [48], oxidative stress and activation of NF-κB [33], prolonged ERK signaling [34], STAT1 signaling [82, 83], and other cytokines including IL-4 and IL-13 [48]. This novel human 3D lung microtissue model captures the complexity of cell-nanomaterial, cell-cell, and cell-matrix interactions induced by exposure to high aspect ratio fibrous particles and provides an alternative to animal models for screening a wide range of diverse nanomaterials [59] and for future mechanistic studies of the complex pathways involved in the development of chronic pulmonary injury and fibrosis.

The observed changes in gene expression are similar to what has been reported following in vivo exposure to comparable materials, linking the molecular changes observed here in 3D human lung microtissues with known biomarkers identified in rodent studies [4, 66, 67]. Both the human fibrosis PCR array and targeted qRT-PCR analysis show consistent changes in genes involved in inflammation and extracellular matrix turnover. Inflammatory cytokines, growth factors, and ECM remodeling genes are highlighted as key mediators involved in fibrogenesis. Paracrine signaling in response to cell damage and cell-nanomaterial interactions is thought to drive the fibrotic response, characterized by complex signaling pathways that result in persistent inflammation, inefficient and excessive unbalanced ECM remodeling, and an unresolved wound healing response [56, 70, 73]. Differentially-expressed genes identified in the PCR array and confirmed using qRT-PCR identify key players in these pathways induced by both MWCNTs and asbestos fibers in 3D microtissues.

While animal studies detect lung fibrosis weeks or months after exposure [25], lung microtissues show morphological and molecular changes in response to nanomaterial exposure after only 4 days. This demonstrates that the 3D lung microtissue model can identify the initial effects of nanomaterial exposure prior to detection of cell damage or loss of viability. Molecular endpoints are not limited to a single biomarker, with MWCNT and asbestos fiber exposure resulting in upregulation of several genes involved in inflammation, fibrogenesis, and ECM remodeling. Key mediators and inflammatory cytokines, including IL-1β, TNF-α, and IL-6 are upregulated both in rodent lungs following in vivo exposure and in the human 3D lung microtissues [5, 36, 51, 56]. Upregulation of similar signaling pathways in both MWCNT-exposed animals and 3D lung microtissues confirms the potential of these novel microtissues as an initial screening tool prior to more expensive chronic rodent inhalation assays [57]. In contrast to crocidolite asbestos, MWCNTs induced more significant alterations in gene expression as well as greater toxicity at equivalent mass doses. This is consistent across both the histopathologic and molecular endpoints, suggesting that the model is able to detect potential pro-fibrotic nanomaterials with relatively high sensitivity and specificity.

Engineered carbon nanotubes are extremely diverse in their production methods, purity, surface modification, dispersion state, biopersistence, shape, and dimensions (reviewed in [84]). All of these parameters including residual metal catalysts, surface functionalization, dispersal, and surface reactivity have been shown to impact their bioactivity as assessed using in vitro screening assays and following short-term in vivo exposures in mice [51, 52, 56, 85, 86]. Physicochemical properties of carbon nanotubes, especially length, diameter, and flexibility have been shown to be critical for uptake by target cells, lysosomal membrane permeabilization, inflammasome activation, and release of mature IL-1β that triggers recruitment of additional inflammatory cells into the lungs [50, 52, 87]. Theoretical modeling studies have been developed to predict potential lung toxicity of high aspect ratio nanomaterials based on their nanomechanical properties [88, 89]. This novel human 3D lung microtissue model was developed using a long, rigid multi-walled carbon nanotube sample that has been widely used to induce acute injury and chronic lung toxicity previously (reviewed in [84]). This 3D microtissue model is useful to validate predictive models and to screen an expanding variety of carbon nanotubes and newer engineered nanomaterials for potential chronic toxicity [51, 57, 89].

## Conclusion

By exploring expression of cytokines and growth factors involved in inflammation and cell-matrix alterations induced by exposure to nanoparticles, this in vitro model can provide an effective screening tool for chronic disease endpoints in the lung. While histopathology qualitatively describes responses like microtissue organization and changes in cell morphology, molecular biomarkers quantify early changes in gene expression at sublethal doses. All three test samples used in this study have been evaluated in previous chronic rodent assays [4, 8, 66, 90] and produce similar responses in human 3D lung microtissues validating this novel platform as a bridging technology between acute in vitro toxicity assays and chronic rodent assays [91, 92]. This novel human 3D lung microtissue has potential application in a tiered testing strategy for detection of potentially fibrogenic nanomaterials [56] and may reduce expensive rodent inhalation studies in assessment of lung toxicity [93].

## Additional file

Additional file 1: **Table S1.** PCR primers. Conditions and sequences used for confirmation of gene expression using qRT-PCR assay for selected targets. **Table S2.** Media formulations. The BEAS-2B epithelial cell media kit from Lonza provided additives required to maintain the health of all three microtissue cell types in 500 mL of high glucose DMEM. Optimized formulations for single cell and microtissue cultures are listed. **Figure S1.** THP-1 material uptake and cathepsin B release. (A) Morphology of undifferentiated THP-1 monocytes and PMA differentiated, un-primed macrophages was observed using brightfield microscopy. (B) LPS-priming promotes phagocytosis by macrophages and material uptake can be observed in primed cells at 24 h after exposure to carbon black, carbon nanotubes, or asbestos fibers. Lysosomal damage and Cathepsin B release following nanomaterial uptake were observed using the Magic Red Cathepsin B Kit according to the protocol described in Zhu et al. 2016 [88]. **Figure S2.** Optimization of microtissue culture. Multiple conditions were tested for the optimization of microtissue formation and maintenance, including the ratio of cell types (A), seeding density (B), and media composition (C). Asterisks indicate areas of necrosis at the center of large microtissues. **Figure S3.** All significantly altered genes altered by exposure to 10 μg/mL of carbon black, carbon nanotubes, and asbestos fibers. This Venn diagram organizes the significantly altered genes (p or q < 0.05) for each exposure, including those shown in the Venn diagram in Fig. 4 and additional statistically significant genes that were up or downregulated less than 2-fold. (DOCX 980 kb).

Additional file 2: PCR array data, 4 day exposure. (XLSX 65 kb)

Additional file 3: PCR array data, 7 day exposure. (XLSX 65 kb)

Additional file 4: Selected PCR array data for analysis. (XLSX 24 kb)
