# Supplementary material for: A novel human 3D lung microtissue model for nanoparticle-induced cell-matrix alterations
Source: Part Fibre Toxicol. 2019 Apr 3;16:15. doi: 10.1186/s12989-019-0298-0 (PMC6448215; doi:10.1186/s12989-019-0298-0)
Supplement: Supplementary file 1 — Table S1. PCR primers. Conditions and sequences used for confirmation of gene expression using qRT-PCR assay for selected targets. Table S2. Media formulations. The BEAS-2B epithelial cell media kit from Lonza provided additives required to maintain the health of all three microtissue cell types in 500 mL of high glucose DMEM. Optimized formulations for single cell and microtissue cultures are listed. Figure S1. THP-1 material uptake and cathepsin B release. (A) Morphology of undifferentiated THP-1 monocytes and PMA differentiated, un-primed macrophages was observed using brightfield microscopy. (B) LPS-priming promotes phagocytosis by macrophages and material uptake can be observed in primed cells at 24 h after exposure to carbon black, carbon nanotubes, or asbestos fibers. Lysosomal damage and Cathepsin B release following nanomaterial uptake were observed using the Magic Red Cathepsin B Kit according to the protocol described in Zhu et al. 2016 [88]. Figure S2. Optimization of microtissue culture. Multiple conditions were tested for the optimization of microtissue formation and maintenance, including the ratio of cell types (A), seeding density (B), and media composition (C). Asterisks indicate areas of necrosis at the center of large microtissues. Figure S3. All significantly altered genes altered by exposure to 10 μg/mL of carbon black, carbon nanotubes, and asbestos fibers. This Venn diagram organizes the significantly altered genes (p or q < 0.05) for each exposure, including those shown in the Venn diagram in Fig. 4 and additional statistically significant genes that were up or downregulated less than 2-fold. (DOCX 980 kb). [file 12989_2019_298_MOESM1_ESM.docx]

**A Novel Human 3D Lung Microtissue Model for Nanoparticle-Induced Cell-Matrix Alterations**

Pranita K. Kabadi^1^, April L. Rodd^1^, Alysha E. Simmons^1^, Norma J. Messier^1^, Robert H. Hurt^2^ and Agnes B. Kane^1^

^1^ Department of Pathology and Laboratory Medicine, ^2^ School of Engineering, Brown University, Providence Rhode Island 02912

Corresponding Authors:

April L. Rodd and Agnes B. Kane

Department of Pathology and Laboratory Medicine

Brown University, Box G-E5

Providence, RI 02912

Tel (401) 863-1110

Fax (401) 863-9008

April_Rodd@brown.edu

Agnes_Kane@brown.edu

Additional file 1

**Table S1: PCR primers.** Conditions and sequences used for confirmation of gene expression using qRT-PCR assay for selected targets.

| Gene | Primer Sequence | qRT-PCR Conditions |
| --- | --- | --- |
| COL1A1 | F: GTGTGGCCCAGAAGAACTGGT  R: CGCCATACTCGAACTGGAATC | 0.2 µM primer  3.0 mM MgCl_2_ |
| COL3A1 | F: GATCAGGCCAGTGGAAATGTA  R: TGTGTTTCGTGCAACCATCC | 0.2 µM primer  2 mM Mg |
| MMP1 | F: CTGTTCTGGGGTGTGGTGTCT  R: CAACCACTGGGCCACTATTTCTC | 0.9 µM primer  2.5 mM MgCl_2_ |
| MMP3 | F: CCATCTCTTCCTTCAGGCGT  R: GTGTGGATGCCTCTTGGGTA | 0.4 µM primer  2.5 mM MgCl_2_ |
| DCN | F: GGCTTCTTATTCGGGTGTGAG R: AGAGCGCACGTAGACACATCT | 0.2 µM primer  2.5 mM Mg |
| HPRT1 | F: GCAGACTTTGCTTTCCTTGG  R: CCAACACTTCGTGGGGTCCTT | 0.2 µM primer  3.0 mM MgCl_2_ |

**Table S2: Media formulations.** The BEAS-2B epithelial cell media kit from Lonza provided additives required to maintain the health of all three microtissue cell types in 500 mL of high glucose DMEM. Optimized formulations for single cell and microtissue cultures are listed.

| **BEAS-2B Epithelial Cell Media: LONZA Clonetics™ BEGM™ BulletKit™ (CC-3170) (single cell culture)** | **Components** | **Volume** |
| --- | --- | --- |
|  | Bronchial Epithelial Cell Basal Medium (BEBM) | 500 mL |
|  | Bovine Pituitary Extract (BPE) | 2 mL |
|  | Hydrocortisone | 0.5 mL |
|  | Human Epidermal Growth Factor (hEGF) | 0.5 mL |
|  | Epinephrine | 0.5 mL |
|  | Transferrin | 0.5 mL |
|  | Insulin | 0.5 mL |
|  | Retinoic Acid | 0.5 mL |
|  | Triiodothyronine | 0.5 mL |
|  | Gentamicin and Amphotericin-B (GA-1000) | 0.5 mL |
| **High FCS Triculture Medium (seeding medium)** | DMEM High Glucose (Thermo Fisher 11965092) | 450 mL |
|  | Heat-inactivated Fetal Calf Serum | 50 mL |
|  | Hydrocortisone | 0.5 mL |
|  | Epinephrine | 0.5 mL |
|  | Transferrin | 0.5 mL |
|  | Insulin | 0.5 mL |
|  | Retinoic Acid | 0.5 mL |
|  | Triiodothyronine | 0.5 mL |
|  | Gentamicin and Amphotericin-B (GA-1000) | 0.5 mL |
| **Low-FCS Triculture Medium (maintenance medium)** | DMEM High Glucose (Thermo Fisher 11965092) | 497.5 mL |
|  | Heat-inactivated Fetal Calf Serum | 2.5 mL |
|  | Hydrocortisone | 0.5 mL |
|  | Epinephrine | 0.5 mL |
|  | Transferrin | 0.5 mL |
|  | Insulin | 0.5 mL |
|  | Retinoic Acid | 0.5 mL |
|  | Triiodothyronine | 0.5 mL |
|  | Gentamicin and Amphotericin-B (GA-1000) | 0.5 mL |
| **THP-1 Monocyte Medium (single cell culture)** | RPMI 1640 (Thermo Fisher 11875119) | 445 mL |
|  | Heat-inactivated Fetal Calf Serum | 50 mL |
|  | Penicillin-Streptomycin (10,000 U/mL) (Thermo Fisher 15140163) | 5 mL |
| **IMR90 Fibroblast Medium (single cell culture)** | DMEM High Glucose (Thermo Fisher 11965092) | 445 mL |
|  | Heat-inactivated Fetal Calf Serum | 50 mL |
|  | Penicillin-Streptomycin (10,000 U/mL) (Thermo Fisher 15140163) | 5 mL |

**Figure S1**

**
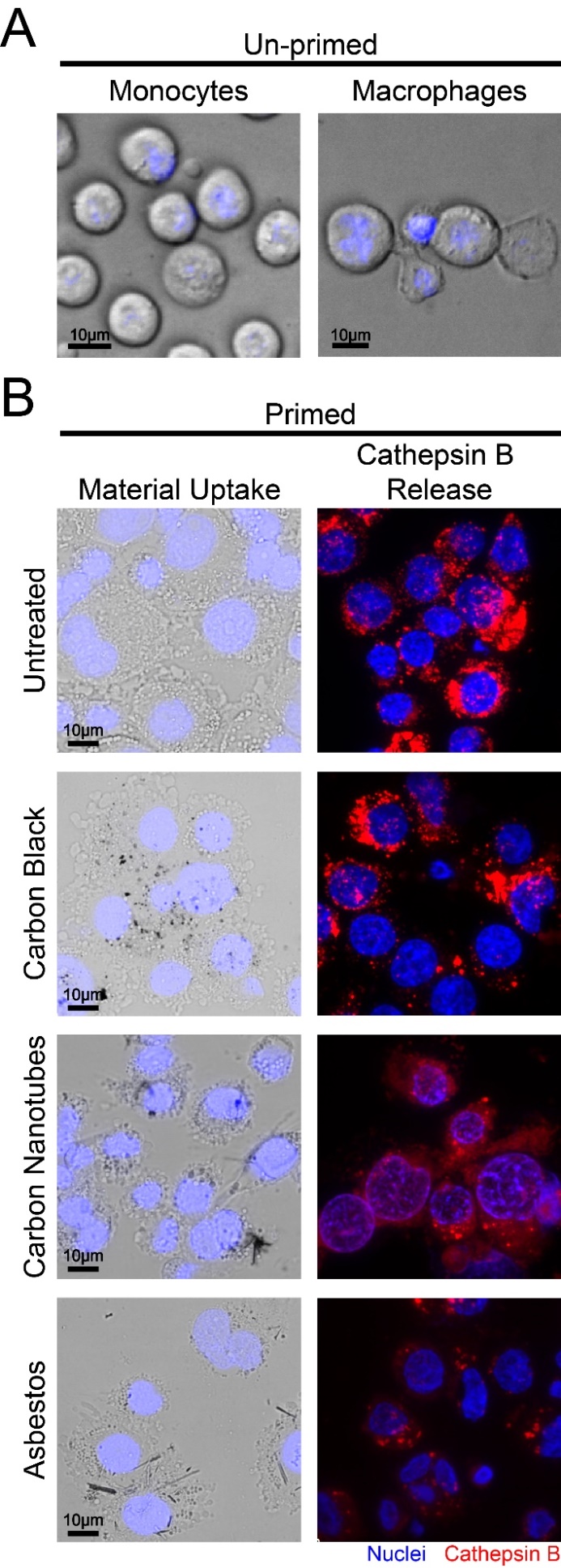
**

**Figure S1: THP-1 material uptake and cathepsin B release.** (A) Morphology of undifferentiated THP-1 monocytes and PMA differentiated, un-primed macrophages was observed using brightfield microscopy. (B) LPS-priming promotes phagocytosis by macrophages and material uptake can be observed in primed cells at 24 hours after exposure to carbon black, carbon nanotubes, or asbestos fibers. Lysosomal damage and Cathepsin B release following nanomaterial uptake were observed using the Magic Red Cathepsin B Kit according to the protocol described in Zhu et al. 2016 [88].

**Figure S2**


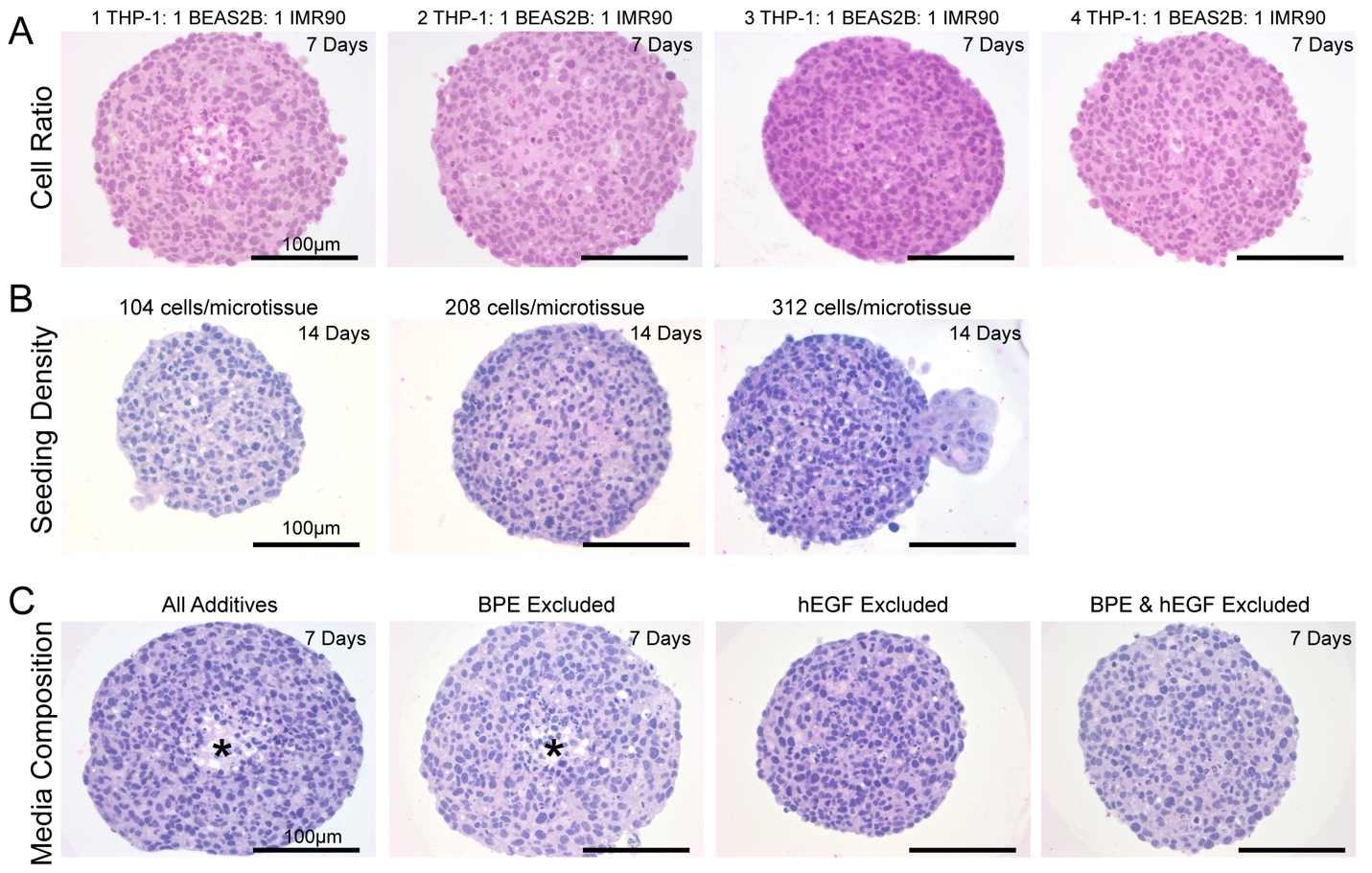


**Figure S2: Optimization of microtissue culture.** Multiple conditions were tested for the optimization of microtissue formation and maintenance, including the ratio of cell types (A), seeding density (B), and media composition (C). Asterisks indicate areas of necrosis at the center of large microtissues.

**Figure S3**

**
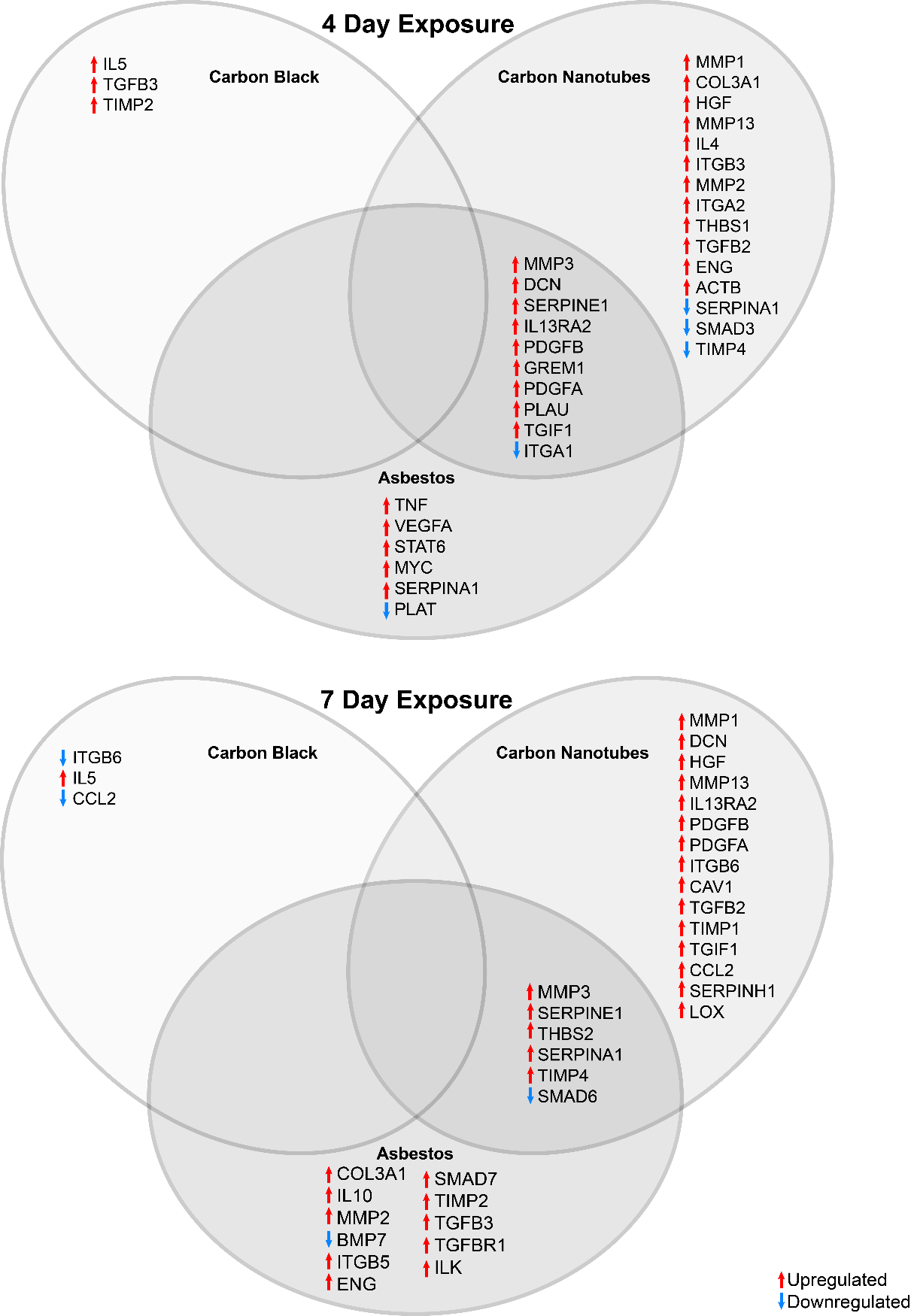
**

**Figure S3: All significantly altered genes altered by exposure to 10μg/mL of carbon black, carbon nanotubes, and asbestos fibers.** This Venn diagram organizes the significantly altered genes (p or q < 0.05) for each exposure, including those shown in the Venn diagram in Figure 4 and additional statistically significant genes that were up or downregulated less than 2-fold.
